# Hesperidin Attenuates Vincristine‐Induced Nephrotoxicity via Nrf2/HO‐1 and PI3K/Akt/mTOR Signaling

**DOI:** 10.1002/jbt.71136

**Published:** 2026-10-18

**Authors:** Aydin Genc, Bayram Akoglan, Emre Sahin, Eren Cankaya

**Affiliations:** ^1^ Department of Biochemistry, Faculty of Veterinary Medicine Bingol University Bingol Türkiye; ^2^ Animal Nutrition and Nutritional Disease Department, Faculty of Veterinary Medicine Bingol University Bingol Türkiye; ^3^ Department of Veterinary Biostatistics, Health Sciences Institute Ankara University Ankara Türkiye; ^4^ Department of Pathology, Faculty of Veterinary Medicine Firat University Elazig Turkey

**Keywords:** apoptosis, hesperidin, nephrotoxicity, oxidative stress, vincristine

## Abstract

Vincristine (VIN) is a widely used chemotherapeutic agent whose therapeutic application is substantially constrained by concentration‐dependent toxicities, including nephrotoxicity. This study aimed to characterize the renoprotective capacity of hesperidin (HES) in a VIN‐induced nephrotoxicity model and to delineate the mechanisms underlying its protective action. Twenty‐eight female Wistar albino rats were divided into four groups: control, HES (200 mg/kg), VIN (0.17 mg/kg), and VIN (0.17 mg/kg) + HES (200 mg/kg), with all treatments administered for 7 consecutive days. Renal function markers, oxidative stress parameters, apoptosis‐ and autophagy‐related gene expression, phosphoinositide 3‐kinase (PI3K)/protein kinase B (Akt)/mammalian target of rapamycin (mTOR) signaling components, and histopathological alterations were evaluated. VIN administration significantly increased serum urea and creatinine levels, lipid peroxidation, and oxidative DNA damage, while reducing antioxidant defenses (*p* < 0.05). VIN also induced marked apoptosis, evidenced by increased tumor protein p53 (p53), Bcl‐2‐associated X protein (Bax), Caspase‐3, and C/EBP homologous protein (CHOP) expression and decreased B‐cell lymphoma 2 (Bcl‐2) levels, together with enhanced autophagy and suppression of PI3K/Akt/mTOR signaling (*p* < 0.05). These molecular changes were accompanied by severe renal histopathological damage (*p* < 0.05). HES supplementation significantly attenuated these effects by restoring antioxidant capacity, reducing inflammatory cytokines, suppressing apoptosis, modulating autophagy‐related gene expression, and partially restoring PI3K/Akt/mTOR signaling (*p* < 0.05). Histopathological findings further confirmed the protective effect of HES against VIN‐induced renal injury (*p* < 0.05). In conclusion, HES exerts renoprotective effects against VIN‐induced nephrotoxicity by modulating interconnected oxidative stress, inflammation, apoptosis, autophagy, and PI3K/Akt/mTOR signaling pathways, suggesting its potential as a multi‐target agent for preventing chemotherapy‐associated renal injury in this experimental model.

## Graphical abstract

Hesperidin attenuated vincristine‐induced nephrotoxicity in rats by improving renal function, antioxidant defenses, and histopathology while suppressing inflammation and apoptosis, modulating autophagy, and partially restoring PI3K/Akt/mTOR signaling. These findings support hesperidin as a promising multi‐target renoprotective adjunct during vincristine chemotherapy.

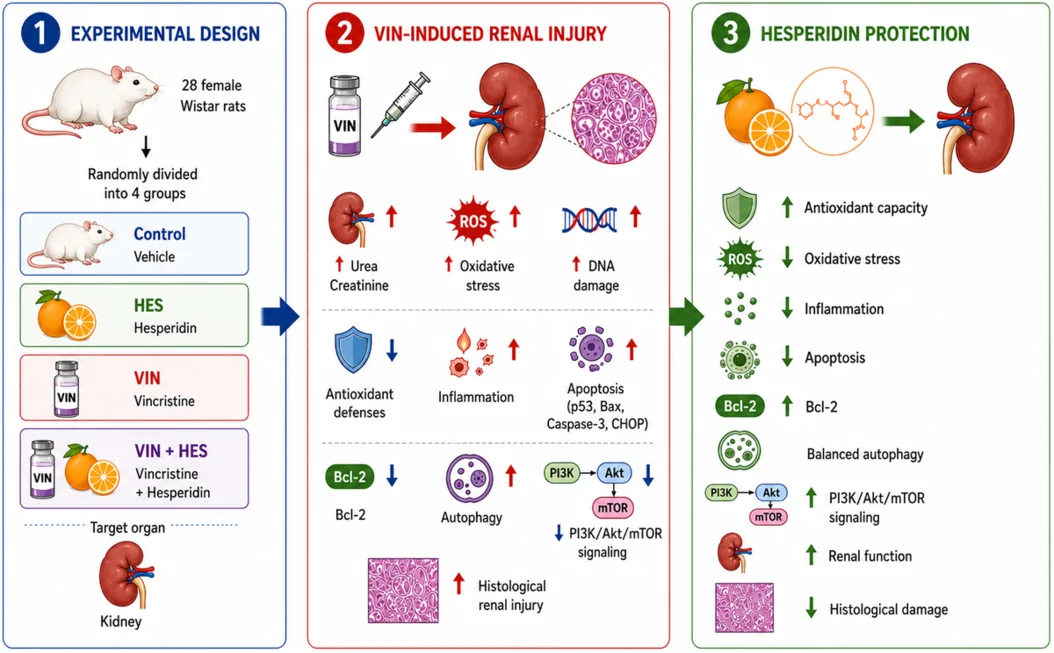

## 1 Introduction

Vincristine (VIN) is a commonly used chemotherapy agent for treating childhood leukemias and various solid tumors [1]. However, its clinical use is limited by dose‐dependent toxicities, including nephrotoxicity. Accumulating experimental evidence indicates that VIN disrupts renal function, as reflected by elevated serum urea and creatinine levels, and perturbs redox homeostasis, thereby promoting inflammatory responses, oxidative stress and apoptotic cell death within renal tissue [2, 3]. These observations underscore the need for adjunctive approaches capable of mitigating renal injury without interfering with the antitumor efficacy of VIN.

Drug‐induced nephrotoxicity is increasingly recognized as a multifactorial process driven by the interplay between oxidative stress, inflammatory activation, and apoptosis. This paradigm has been consistently demonstrated across diverse nephrotoxic models, including those induced by chemotherapeutic agents and nonsteroidal anti‐inflammatory [4, 5, 6]. Excessive generation of reactive oxygen species (ROS), accompanied by lipid peroxidation and depletion of endogenous antioxidant systems, such as glutathione (GSH), superoxide dismutase (SOD), and catalase (CAT), contributes to the amplification of inflammatory signaling and the activation of mitochondria‐dependent apoptotic pathways in kidney [4, 6, 7]. Accordingly, therapeutic strategies targeting these interconnected processes are considered promising for the prevention of nephrotoxicity.

Hesperidin (HES), a bioactive flavonoid abundantly present in citrus fruits, has gained attention due to its demonstrated antioxidant, antinflammatory, and antiapoptotic activities in multiple models of renal injury [6, 8]. In experimental models of cisplatin and cyclosporine‐induced nephrotoxicity, HES has been shown to improve renal function, restore antioxidant defense's, and attenuate inflammatory responses, partly through suppression of key mediators such as tumor necrosis factor‐α (TNF‐α) and nuclear factor kappa‐light‐chain‐enhancer of activated B cells, alongside modulation of apoptotic signaling pathways, including downregulation of Bcl‐2‐associated X protein (Bax) and Caspase‐3 and upregulation of B‐cell lymphoma 2 (Bcl‐2) [6, 8, 9]. In addition, HES and its derivatives have been reported to enhance cellular resilience against oxidative injury via activation of redox‐sensitive pathways, including Nrf2/HO‐1 signaling [6, 10].

Despite the reported renoprotective effects of hesperidin in various experimental models, evidence regarding its protective role against vincristine‐induced nephrotoxicity remains limited, and the underlying mechanisms have not yet been fully elucidated.

## 2 Material and Methods

### 2.1 Drugs and Chemicals

Koçak Pharma (Istanbul, Türkiye) provided vincristine sulfate as a sterile injectable solution in a vial (2 mg/2 mL), while Sigma‐Aldrich (St. Louis, MO, USA) provided hesperidin (> 80% purity, powder). All other chemicals and reagents used in this study were of analytical grade.

### 2.2 Animals and Ethical Approval

A total of 28 female Wistar albino rats (8–9 weeks old; 180–200 g body weight) obtained from Bingol University's Experimental Research Center were used. All animals were acclimatized for 1 week prior to the experiment under standard laboratory conditions (24°C ± 1°C, 50% ± 5% humidity, 12 h light/dark cycle). The animals were kept in normal cages with unlimited access to drinking water and a commercial pellet feed during the study period. Approval for the experimental protocols involving animals was obtained from the Animal Experiments Ethics Committee of Bingol University (Protocol No: E‐85680299‐020‐2600023988). The procedures were carried out in compliance with accepted ethical standards and reported following the ARRIVE guidelines.

### 2.3 Experimental Design

The experimental animals were randomly divided into four groups, with seven rats in each group. All treatments were administered once daily for 7 consecutive days. The HES dose (200 mg/kg) was selected based on the study by Ileriturk et al. [11], whereas the VIN dosing regimen was adapted from Khalilzadeh et al. [12] and modified according to the experimental design of the present study.

1‐Control: Rats received intraperitoneal injections of normal saline once daily for 7 days.

2‐HES: HES was dissolved in normal saline and administered orally at a dose of 200 mg/kg once daily for 7 days.

3‐VIN: Rats received VIN intraperitoneally at a dose of 0.17 mg/kg once daily for 7 days, resulting in a total cumulative dose of approximately 1.2 mg/kg.

4‐VIN + HES: Rats received HES, dissolved in normal saline, orally at a dose of 200 mg/kg once daily for 7 days, together with intraperitoneal VIN at a dose of 0.17 mg/kg once daily for 7 days. The total cumulative VIN dose was approximately 1.2 mg/kg.

The rats were beheaded under mild sevoflurane anesthesia 24 h after the last VIN dose (Day 8). Blood samples were collected for serum separation, and kidney tissues were excised for subsequent analyses. While the remaining samples were stored at −80°C until further analysis by Western blotting and real‐time PCR, a portion of the kidney tissues was fixed in 10% formaldehyde for histological assessment.

### 2.4 Kidney Function Tests

Serum urea and creatinine levels were determined using commercially available Randox assay kits (urea, SKU: UR3825; creatinine, SKU: CR8316) on a Randox RX Series automated biochemical analyzer, as previously described [13].

### 2.5 Determination of Oxidative Stress and Antioxidant Parameters

Homogenization and determination procedures were carried out as previously described for MDA, GSH, GPx, SOD, and CAT [14]. Lipid peroxidation (MDA), SOD, CAT, GPx, and GSH levels were determined according to Placer et al. [15], Sun et al. [16], Aebi [17], Matkovics et al. [18], and Sedlak and Lindsay [19], respectively. Protein concentrations were quantified following the method of Lowry et al. [20].

### 2.6 ELISA‐Based PI3K/Akt/mTOR and 8‐OHdG Analysis

The levels of 8‐hydroxy‐2′‐deoxyguanosine (8‐OHdG), phosphoinositide 3‐kinase (PI3K), protein kinase B (Akt), and mammalian target of rapamycin (mTOR) were measured by homogenizing kidney tissue samples in 0.1 M phosphate buffer (pH 7.4) using a TissueLyser instrument (TissueLyser II, Qiagen). The supernatants were collected for examination after the homogenates were centrifuged. The concentrations of 8‐OHdG (201‐11‐0032), PI3K (201‐11‐0439), Akt (201‐11‐0202), and mTOR (201‐11‐0427) were evaluated in accordance with the manufacturer's instructions using commercially available ELISA assays (Sunred Biological Technology, Shanghai, China). The optical density at 450 nm was measured using a microplate reader (BioTek, Winooski, VT, USA). The results were determined by employing standard calibration curves.

### 2.7 Real‐Time Quantitative PCR Analysis in Kidney Tissue

The mRNA expression levels of the following genes were analyzed using gene‐specific primers, as specified in Table 1: tumor protein p53 (p53), Bax, Bcl‐2, Caspase‐3, C/EBP homologous protein (CHOP), microtubule‐associated protein 1 light chain 3 (LC3B), Beclin‐1, autophagy‐related gene 5 (ATG5), and autophagy‐related gene 7 (ATG7). Total RNA was extracted from tissue samples using QIAzol Lysis Reagent (Qiagen, Germany; Cat. No. 79306). The RNA concentrations were standardized across samples, and the RNA purity and quantity were assessed using a NanoDrop spectrophotometer (BioTek Instruments, EPOCH, USA). In accordance with the manufacturer's instructions, the iScript cDNA Synthesis Kit (Bio‐Rad, USA) was employed to generate complementary DNA (cDNA). The qRT‐PCR was conducted using a Rotor‐Gene Q system (Qiagen, Germany) and iTaq Universal SYBR Green Supermix (Bio‐Rad, USA). Every reaction was conducted in triplicate, with GAPDH serving as the internal reference gene. The relative mRNA expression was determined using Livak and Schmittgen's 2^−^^ΔΔCt^ technique [21].

**Table 1:** Primer sequences.

Gene	Sequences (5′–3′)	Length (bp)	Accession no.
p53	F: GCGCTTCGAGATGTTCCGA R: AGACTGGCCCTTCTTGGTCT	121	NM_030989.3
Bax	F: TCATCCAGGATCGAGCAG R: ATCATCCTCTGCAGCTCCA	154	NM_017059.2
Bcl‐2	F: ACTTTGCAGAGATGTCCAG R: CAGGTACTCAGTCATCCAC	214	NM_016993.2
Caspase‐3	F: CTGGAATGTCAGCTCGCAA R: CAGTAGTCGCCTCTGAAGA	270	NM_012922.2
CHOP	F: GAAGCCTGGTATGAGGATCT R: GAACTCTGACTGGAATCTGG	209	NM_001109986.1
LC3B	F: GAGCTTCGAACAAAGAGTGG R: CGCTCATATTCACGTGATCA	152	NM_022867.2
Beclin‐1	F: TCTCGTCAAGGCGTCACTTC R: CCATTCTTTAGGCCCCGACG	198	NM_053739.2
ATG5	F: CTCTGCCTTGGAACATCACA R: ATCATTCTGCAGTCCCATCC	135	NM_001014250
ATG7	F: ATTGCTGCATCAAGAAACCC R: GATGGAGAGCTCCTCAGCA	142	NM_001012097
GAPDH	F: AGTATGTCGTGGAGTCTAC R: AGGATGCATTGCTGACAAT	179	NM_017008.4

### 2.8 Western Blot Analysis

HO‐1, Nrf2, TNF‐α, and IL‐1β levels in kidney tissue were measured by Western blot utilizing a Trans‐Blot Turbo transfer unit (Bio‐Rad, Hercules, CA, USA) and a Mini‐PROTEAN Tetra Vertical Electrophoresis system [22]. After homogenizing tissue samples in a suitable lysis solution with protease inhibitors, the homogenates were kept at −80°C until analysis. After determining the total protein concentrations, equivalent amounts of protein were separated on 12% SDS–polyacrylamide gels and then transferred onto nitrocellulose membranes. Following blocking, membranes were incubated for a whole night at 4°C with particular primary antibodies and then with the corresponding secondary antibodies. Chromogenic detection was used to visualize immunoreactive bands. Densitometric analysis was used to quantify band intensities using ImageJ software. Protein expression levels were represented as relative fold changes in comparison to the control group after being normalized to β‐actin as a loading control.

### 2.9 Histopathological Analysis

An automated tissue processor was used to fix kidney tissues in 10% neutral buffered formalin, process them through a graded ethanol series, clear them in xylene, and embed them in paraffin. After standard processing, an embedding station (Leica EG 1150 H, Wetzlar, Germany) was used to embed the specimens in paraffin blocks. A rotary microtome (Leica RM2125, Wetzlar, Germany) was used to segment paraffin‐embedded tissues at a thickness of 3–5 µm. The sections were then placed on positively charged slides. Using an automated staining equipment (Leica Autostainer XL, Wetzlar, Germany), the sections were stained with H&E in accordance with the procedure outlined by Luna [23]. A light microscope was used for the histopathological examination, and sample photomicrographs were taken.

Histopathological assessment of the kidney tissues was performed using a semiquantitative method. The evaluation criteria included tubular necrosis, loss of tubular epithelial cells, formation of necrotic tubular hyaline casts, and tubular dilatation. Histopathological alterations in the kidney tissues were graded using a semiquantitative scoring system based on the extent of damage in the cortex and medulla, according to previously defined criteria (Table 2) [24].

**Table 2:** Kidney damage assessment chart.

Score	Histopathological damage
0	Normal histological structure
0.5	Histopathological damage: < 10%
1	Histopathological damage: 10%–25%
2	Histopathological damage: 25%–50%
3	Histopathological damage: 50%–75%
4	Histopathological damage: 75%–100%

### 2.10 Statistical Analysis

The Shapiro–Wilk test was used to assess data normality, and Levene's test was used to evaluate the homogeneity of variances. For normally distributed data with homogeneous variances, group differences were analyzed using one‐way analysis of variance (ANOVA), followed by Tukey's HSD post‐hoc test. When the assumption of normality was satisfied but homogeneity of variances was violated, Welch's ANOVA followed by the Games–Howell post‐hoc test was applied. Non‐normally distributed data were analyzed using the Kruskal–Wallis test followed by Dunn's multiple‐comparison test. Histopathological scores were considered ordinal data and were therefore analyzed using the Kruskal–Wallis test followed by Dunn's multiple‐comparison test. Parametric data are presented as mean ± standard error of the mean (SEM), whereas non‐parametric and ordinal data are presented as median (interquartile range). IBM SPSS Statistics version 22.0 [25] was used for all statistical analyses, and *p* < 0.05 was considered statistically significant.

## 3 Results

### 3.1 Serum Urea and Creatinine Levels

As shown in Figure 1, serum urea and creatinine degrees differed significantly among the experimental groups (*p* < 0.05). In the VIN group, both urea and creatinine degrees were markedly elevated compared to the control and HES groups (*p* < 0.05), indicating impaired renal function. In contrast, no significant difference was observed between the control and HES groups for either parameter (*p* > 0.05). HES treatment in combination with VIN significantly reduced both urea and creatinine levels compared to the VIN group (*p* < 0.05). However, these values remained higher than those of the control group (*p* < 0.05), indicating that HES treatment partially attenuated VIN‐induced renal damage.

**Figure 1:**
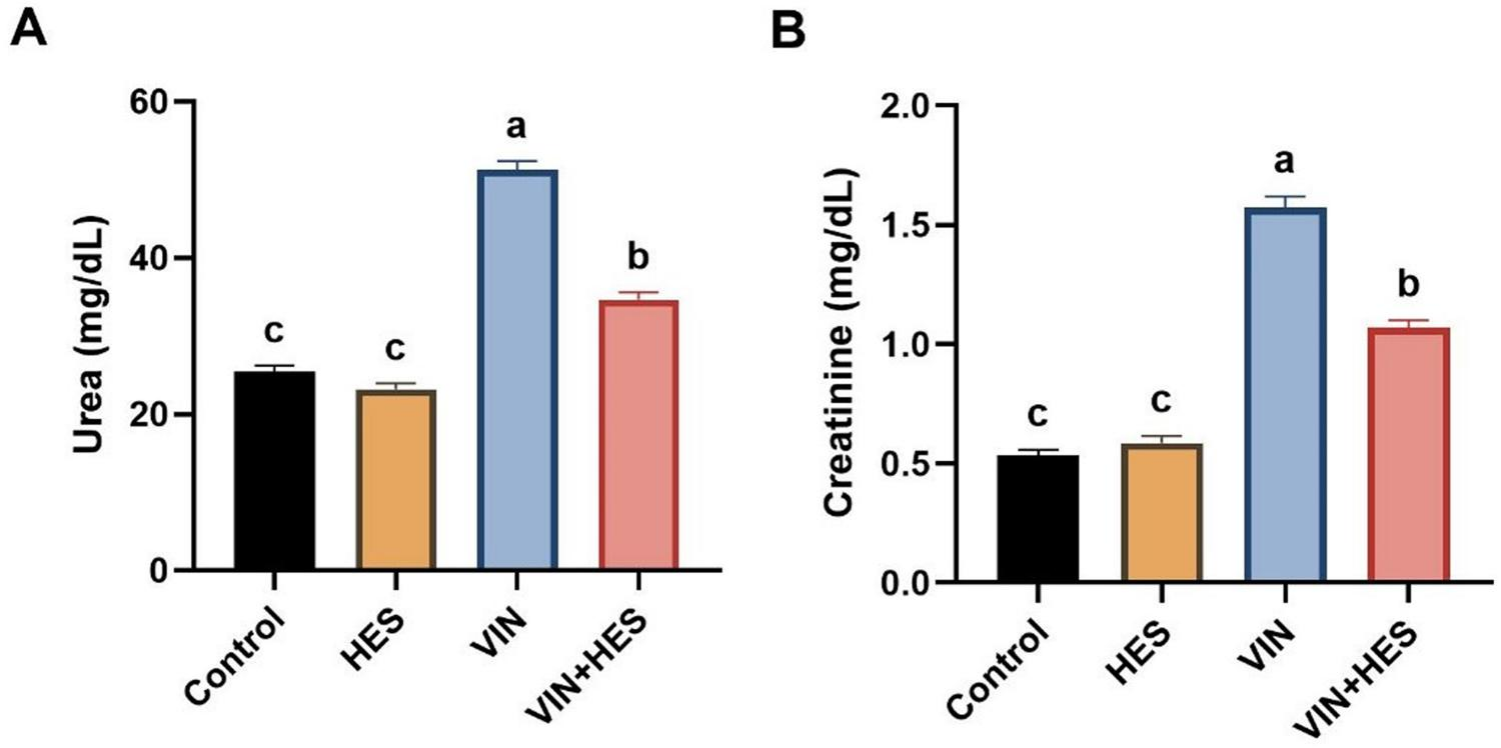
Effects of VIN and HES treatment on rat serum levels of urea (A) and creatinine (B). The mean ± SEM was used to express data. Data were normally distributed and analyzed using one‐way ANOVA followed by Tukey's HSD test. Different letters (a, b, c) above columns denote statistically significant differences between groups (*p* < 0.05). Abbreviations: HES, hesperidin; VIN, vincristine.

### 3.2 Analysis of DNA Damage and Oxidative Stress Markers in Kidney

As shown in Figure 2, oxidative stress and antioxidant defense parameters differed significantly among the experimental groups (*p* < 0.05). VIN exposure led to a pronounced elevation in MDA and 8‐OHdG levels compared with both the control and HES groups (*p* < 0.05), reflecting increased lipid peroxidation and oxidative DNA damage. Conversely, antioxidant indicators, GSH, SOD, CAT, and GPx were significantly reduced in the VIN‐treated group relative to control and HES (*p* < 0.05). Co‐administration of HES in the VIN + HES group mitigated these disturbances. Specifically, MDA and 8‐OHdG levels were significantly lower than those observed in the VIN group (*p* < 0.05), whereas GSH, SOD, CAT, and GPx activities were significantly elevated (*p* < 0.05). Despite these improvements, the measured parameters did not reach baseline control values, suggesting that HES confers only partial protection against VIN‐induced oxidative imbalance. No statistically significant differences were detected between the control and HES groups for any of the evaluated parameters (*p* > 0.05).

**Figure 2:**
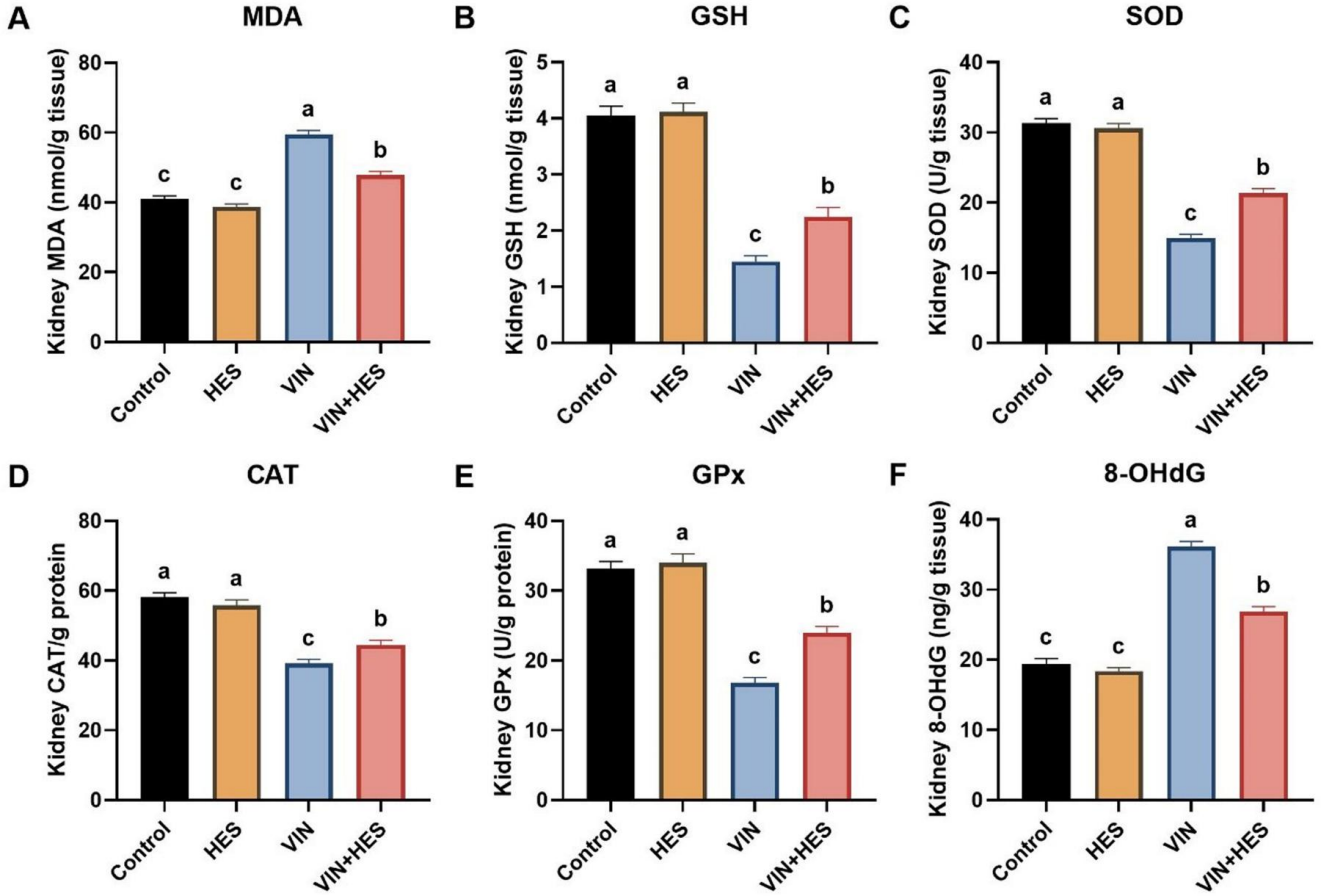
Rat kidney tissue oxidative stress and antioxidant properties after VIN and HES treatment. The experimental groups' levels of MDA (A), GSH (B), SOD (C), CAT (D), GPx (E), and 8‐OHdG (F). The mean ± SEM was used to express data. Data were normally distributed and analyzed using one‐way ANOVA followed by Tukey's HSD test. Different letters (a, b, c) above columns denote statistically significant differences between groups (*p* < 0.05). Abbreviations: 8‐OHdG, 8‐hydroxy‐2′‐deoxyguanosine; CAT, catalase; GPx, glutathione peroxidase; GSH, glutathione; HES, hesperidin; MDA, malondialdehyde; SOD, superoxide dismutase; VIN, vincristine.

### 3.3 Effects of VIN and HES on Apoptosis and Inflammation‐Related Gene Expression in Rat Kidney Tissue

As illustrated in Figure 3, apoptosis and stress‐associated gene expression profiles showed significant differences among the experimental groups (*p* < 0.05). VIN treatment resulted in a marked upregulation of pro‐apoptotic and stress‐related genes, including p53, Bax, Caspase‐3, and CHOP, compared with the control and HES groups (*p* < 0.05), suggesting activation of apoptotic signaling and endoplasmic reticulum stress. In contrast, the expression of the anti‐apoptotic gene Bcl‐2 was significantly downregulated in the VIN group relative to control and HES (*p* < 0.05). Co‐treatment with HES in the VIN + HES group mitigated these effects. Specifically, the elevated mRNA levels of p53, Bax, Caspase‐3, and CHOP were significantly decreased compared to the VIN group (*p* < 0.05), whereas Bcl‐2 expression was significantly restored (*p* < 0.05). Nevertheless, these parameters did not fully reach control values, indicating that HES exerts a partial protective effect against VIN‐induced apoptosis and cellular stress. There are no significant differences detected between the control and HES groups in terms of p53, Bax, Caspase‐3, and CHOP expression (*p* > 0.05).

**Figure 3:**
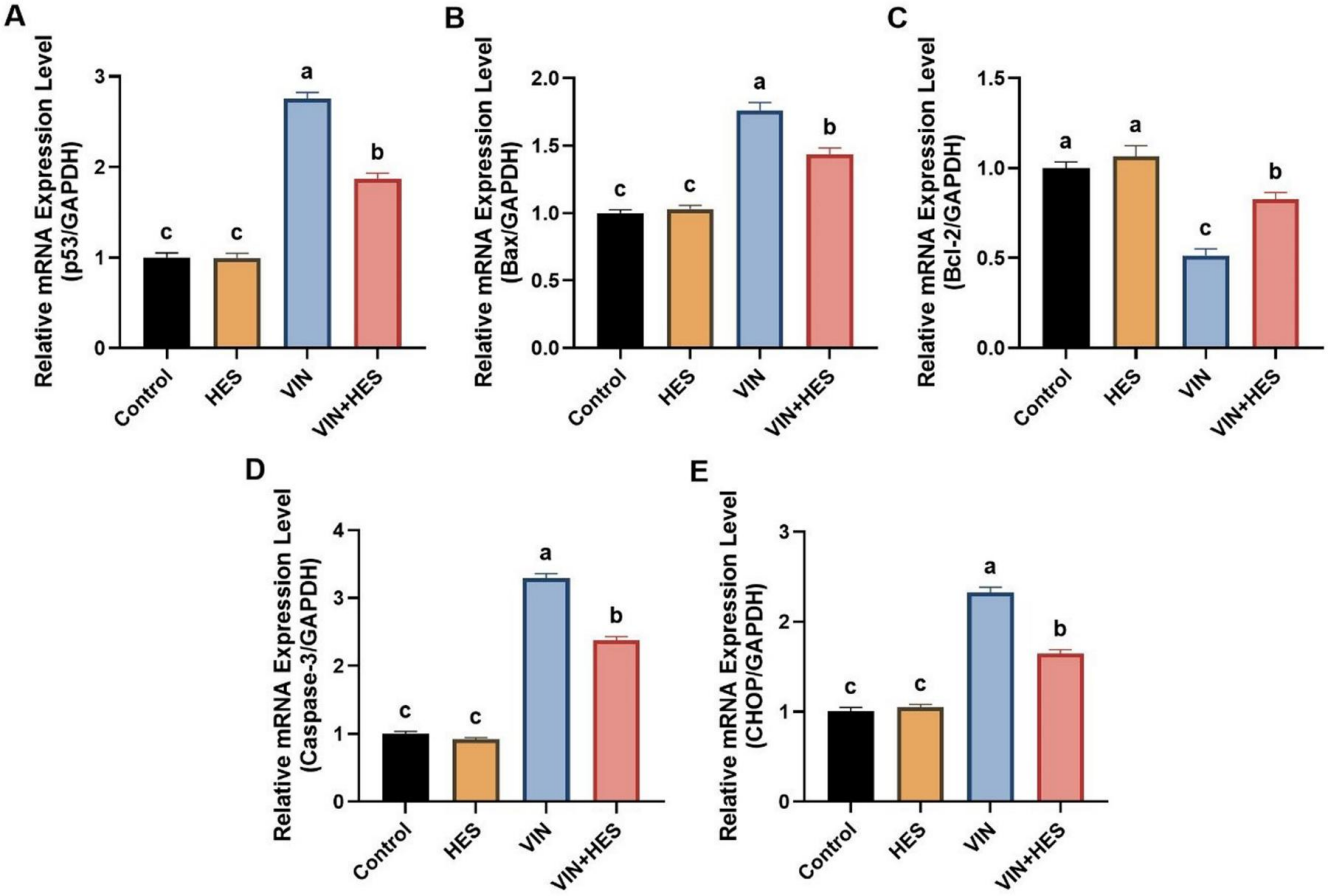
Effects of VIN and HES treatment on the expression of genes linked to stress and apoptosis in rat kidney tissue. mRNA expression levels in the experimental groups for p53 (A), Bax (B), Bcl‐2 (C), Caspase‐3 (D), and CHOP (E). The mean ± SEM was used to express data. Data did not meet the normality assumption and were analyzed using the Kruskal–Wallis test followed by Dunn's multiple‐comparison test. Different letters (a, b, c) above columns denote statistically significant differences between groups (*p* < 0.05). Abbreviations: Bax, Bcl‐2‐associated X protein; Bcl‐2, B‐cell lymphoma 2; CHOP, C/EBP homologous protein; HES, hesperidin; VIN, vincristine; p53, tumor protein p53.

### 3.4 Autophagy‐Related Gene Expression in VIN‐Induced Renal Injury

As shown in Figure 4, VIN exposure significantly upregulated the mRNA expression of autophagy‐related genes in kidney tissue (*p* < 0.05). Specifically, LC3B, Beclin‐1, ATG5, and ATG7 expression levels were markedly higher in the VIN group compared with the control and HES groups (*p* < 0.05), reflecting activation of the autophagic machinery. Co‐administration of HES (VIN + HES) mitigated these elevations, as the expression levels of LC3B, Beclin‐1, ATG5, and ATG7 were significantly lower than those observed in the VIN group (*p* < 0.05). Nevertheless, these values remained significantly above control levels (*p* < 0.05), indicating that HES exerts a partial modulatory effect on VIN induced autophagy.

**Figure 4:**
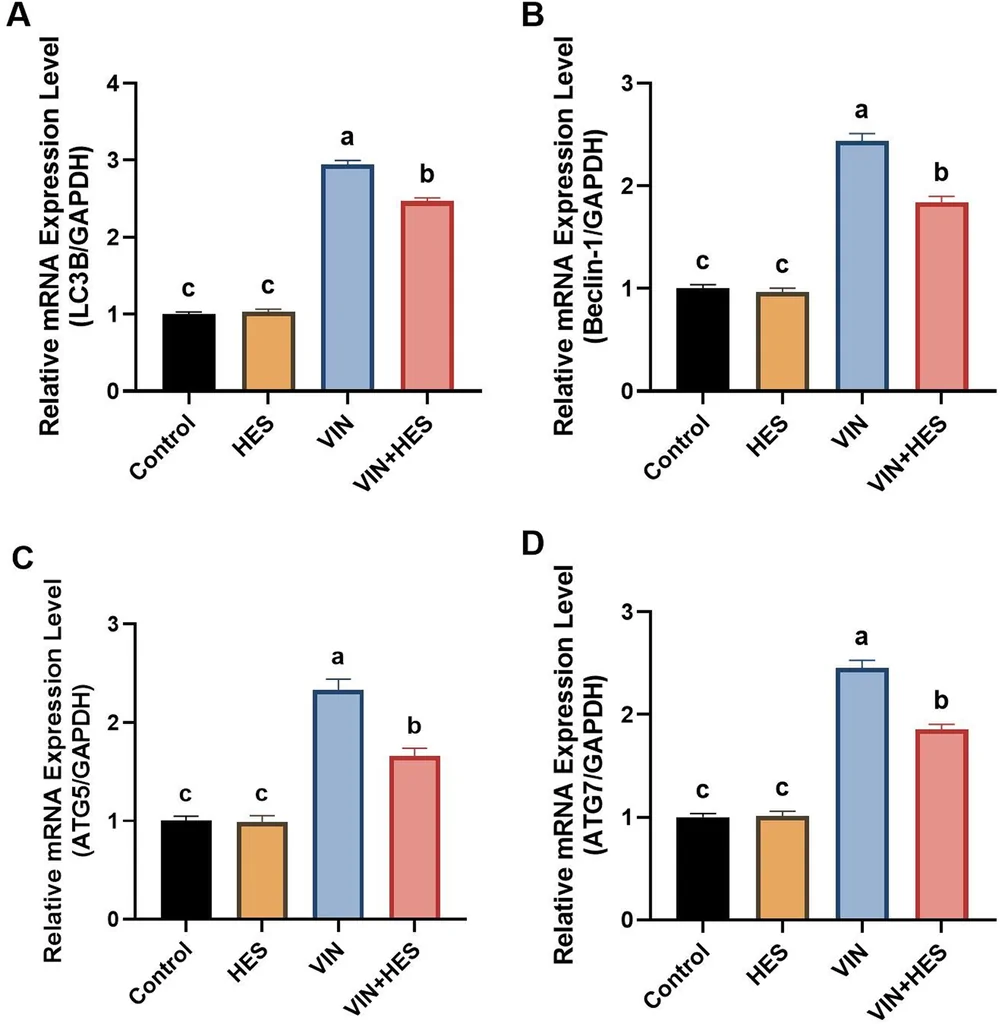
Effects of VIN and HES treatments on the expression of genes linked to autophagy in rat kidney tissue. The experimental groups' mRNA expression levels for LC3B (A), Beclin‐1 (B), ATG5 (C), and ATG7 (D). The mean ± SEM was used to express data. Data did not meet the normality assumption and were analyzed using the Kruskal–Wallis test followed by Dunn's multiple‐comparison test. Different letters (a, b, c) above columns denote statistically significant differences between groups (*p* < 0.05). Abbreviations: ATG5, autophagy‐related gene 5; ATG7, autophagy‐related gene 7; HES, hesperidin; LC3B, microtubule‐associated protein 1 light chain 3; VIN, vincristine.

### 3.5 PI3K/Akt/mTOR Pathway Alterations in VIN‐Induced Renal Injury

As shown in Figure 5, significant alterations were observed in PI3K/Akt/mTOR pathway components among the experimental groups (*p* < 0.05). VIN administration significantly decreased PI3K, Akt, and mTOR levels compared to the control and HES groups (*p* < 0.05), indicating suppression of this signaling pathway. HES administration alone did not produce significant changes compared to the control group for any of the evaluated parameters (*p* > 0.05). However, HES supplementation in the VIN + HES group significantly attenuated these alterations. Specifically, PI3K, Akt, and mTOR levels were significantly increased compared to the VIN group (*p* < 0.05), although they did not fully return to control levels. These findings suggest that HES partially restores VIN‐induced suppression of the PI3K/Akt/mTOR signaling pathway.

**Figure 5:**
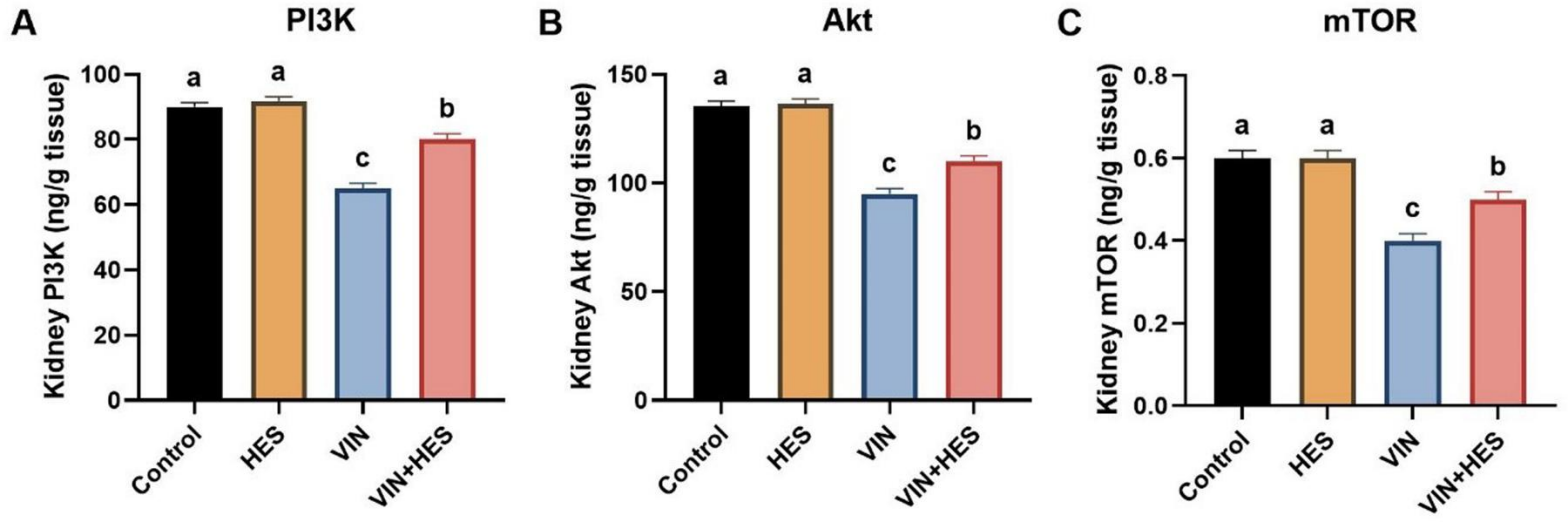
PI3K/Akt/mTOR pathway components in rat kidney tissue are affected by VIN and HES treatment. The experimental groups' levels of PI3K (A), Akt (B), and mTOR (C). The mean ± SEM was used to express data. Data were normally distributed and analyzed using one‐way ANOVA followed by Tukey's HSD test. Different letters (a, b, c) above columns denote statistically significant differences between groups (*p* < 0.05). Abbreviations: Akt, protein kinase B; HES, hesperidin; mTOR, mammalian target of rapamycin; PI3K, phosphoinositide 3‐kinase; VIN, vincristine.

### 3.6 Effects of VIN and HES on Nrf2/HO‐1 Signaling and Inflammatory Markers in Rat Kidney Tissue

As presented in Figure 6, significant alterations were observed in Nrf2/HO‐1 signaling and inflammatory markers among the experimental groups (*p* < 0.05). VIN administration markedly decreased HO‐1 and Nrf2 protein levels compared to the control and HES groups (*p* < 0.05), indicating suppression of the antioxidant defense system. In contrast, pro‐inflammatory cytokines TNF‐α and IL‐1β were significantly increased in the VIN group relative to control and HES groups (*p* < 0.05). HES administration alone significantly increased HO‐1 and Nrf2 levels compared to the control group (*p* < 0.05), while TNF‐α and IL‐1β levels remained comparable to control (*p* > 0.05). HES supplementation with VIN (VIN + HES) significantly attenuated these alterations. Specifically, HO‐1 and Nrf2 levels were significantly increased compared to the VIN group (*p* < 0.05), whereas TNF‐α and IL‐1β levels were significantly reduced (*p* < 0.05). However, these parameters did not fully return to control levels. These findings were further supported by Western blot analysis, which showed consistent changes in protein expression levels. Overall, the results indicate that HES mitigates VIN‐induced oxidative stress and inflammatory responses, at least in part, through modulation of the Nrf2/HO‐1 signaling pathway.

**Figure 6:**
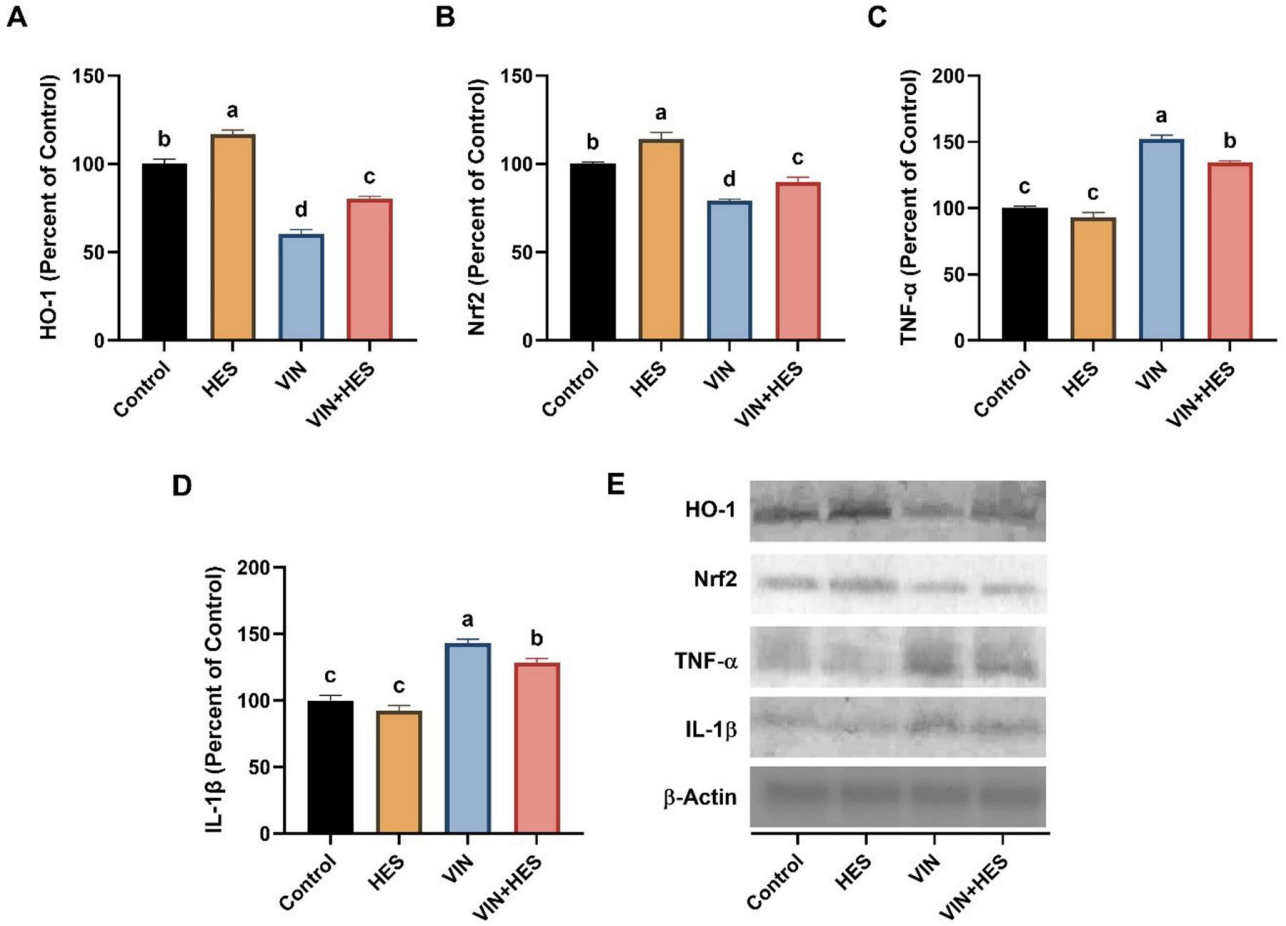
VIN and HES effects on inflammatory markers and Nrf2/HO‐1 signaling in rat kidney tissue. Levels of HO‐1 (A), Nrf2 (B), TNF‐α (C), IL‐1β (D), and representative Western blot pictures (E) of the experimental groups. All results were displayed as mean ± SEM and represented as percent of control (A–D). Data did not meet the normality assumption and were analyzed using the Kruskal–Wallis test followed by Dunn's multiple‐comparison test. Different letters (a, b, c, d) above columns denote statistically significant differences between groups (*p* < 0.05). Abbreviations: HES, hesperidin; HO‐1, heme oxygenase‐1; IL‐1β, interleukin‐1β; Nrf2, nuclear factor erythroid 2–related factor 2; TNF‐α, tumor necrosis factor‐α; VIN, vincristine. The full Western blot images are shown in Figure S1.

### 3.7 Histopathological Findings

In the Control (A), renal sections exhibited normal histomorphological architecture, with well‐defined glomerular and tubular structures, as illustrated in Figure 7. Glomeruli demonstrated intact capillary loops, normal Bowman's space, and preserved tubular epithelial integrity. In the HES group (B), the overall histological architecture of the renal parenchyma was maintained. Glomeruli retained normal morphology, and tubular organization remained intact, with no evident degenerative alterations. In the VIN group (C), pronounced histopathological alterations were observed. Glomeruli appeared atrophic and necrotic, accompanied by marked interstitial congestion. Tubular structures exhibited evident degeneration, characterized by epithelial cell damage, loss of structural integrity, and hyaline cast formation within the lumina. These findings indicate severe cellular injury and structural disruption in the renal tissue. In the VIN + HES group (D), a marked attenuation of histopathological damage was observed compared to the VIN group. Glomerular structures were largely preserved, interstitial congestion was reduced, and tubular degenerative changes were substantially alleviated. Notably, only a limited number of atrophic tubules persisted. Overall, these findings suggest a protective effect of HES against VIN‐induced renal injury.

**Figure 7:**
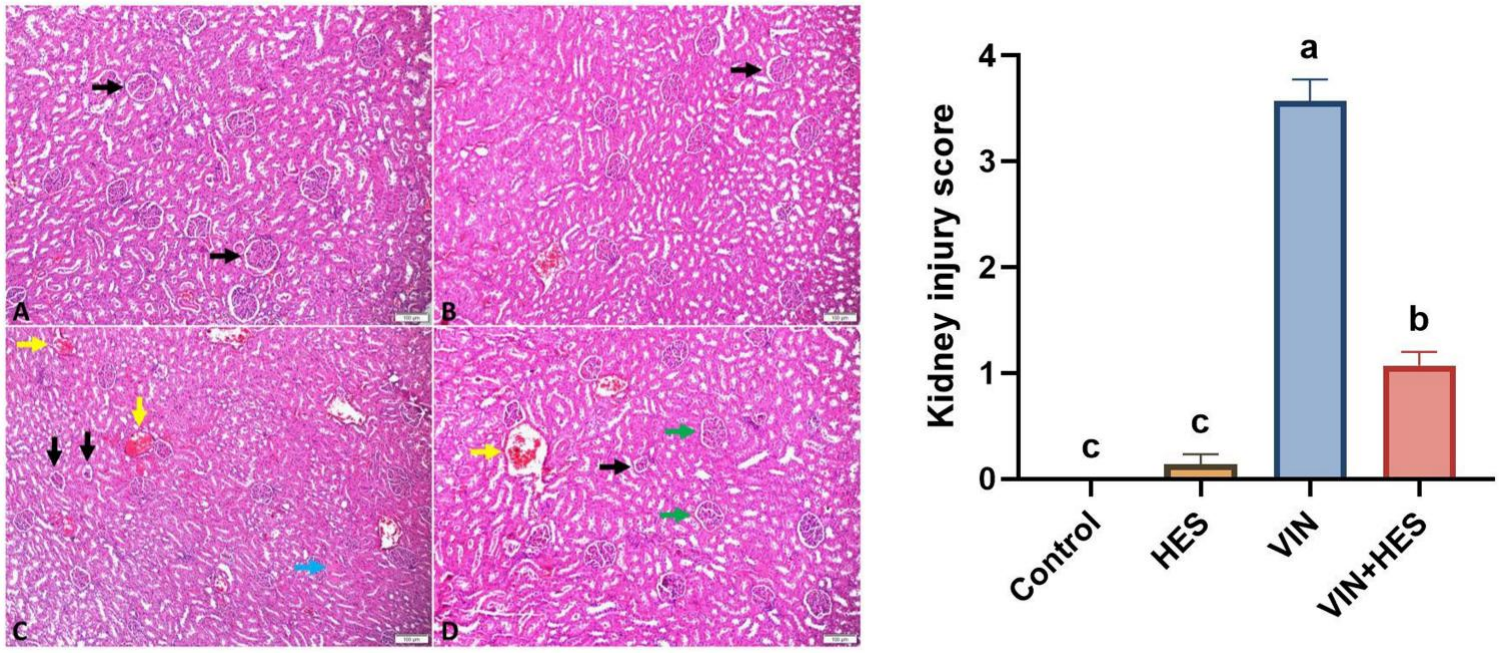
Histopathological evaluation of kidney tissue and injury scoring. In the control group (A), glomeruli (black arrow) exhibit normal histomorphological structure. In the HES group (B), the overall histological architecture of the renal parenchyma is preserved, and glomeruli display regular morphology (black arrow). In the VIN group (C), atrophic and necrotic glomeruli (black arrow), congestion in the interstitial areas (yellow arrow), and degenerative changes in tubular structures, along with hyaline cast accumulation, are observed (blue arrow). In the VIN + HES group (D), glomerular structures are largely preserved (green arrow), only a single atrophic tubule is present (black arrow), and congestion is markedly reduced compared to the vincristine group (yellow arrow). (H&E; scale bar = 100 µm; magnification = 10×). Histopathological scores were treated as ordinal data and analyzed using the Kruskal–Wallis test followed by Dunn's multiple‐comparison test. Data are presented as median (IQR). Different letters in the columns (a,b,c) indicate statistically significant differences between groups (*p* < 0.05). Abbreviations: HES, hesperidin; VIN, vincristine.

## 4 Discussion

The present study demonstrates that VIN induces significant renal injury characterized by inflammation, oxidative stress, apoptosis, and dysregulation of autophagy and PI3K/Akt/mTOR signaling pathways. Importantly, HES supplementation partially attenuated these alterations, suggesting a multi‐target protective effect.

Many experimental studies and clinical investigations use serum urea and creatinine levels as established biomarkers for assessing renal function [1, 3]. In line with our findings, VIN has been reported to significantly elevate serum urea and creatinine levels in rats, indicating impaired renal function and nephrotoxicity [3]. Consistent with our findings, HES has been reported to reduce elevated serum urea and creatinine levels, indicating improved renal function [26, 27].

VIN‐induced nephrotoxicity is primarily characterized by a disruption of redox homeostasis resulting from excessive generation of ROS. Consistent with previous reports, our findings demonstrated marked increases in lipid peroxidation and oxidative DNA damage markers, including MDA and 8‐OHdG, accompanied by concomitant decreases in key antioxidant defense systems such as SOD, CAT, and GPx [2, 3, 28]. This pattern may be attributed to the excessive consumption of these enzymatic antioxidants, along with reduced GSH, during the detoxification of VIN‐induced ROS. Our results are in agreement with this profile and further support the central role of oxidative stress in VIN‐associated renal injury. Furthermore, the elevation of 8‐OHdG levels reflects the accumulation of oxidative DNA lesions, suggesting a potential compromise in genomic stability under sustained oxidative stress conditions [29]. Consistent with previous reports, HES has been shown to restore antioxidant defense systems (SOD, CAT, GPx, and GSH) and attenuate oxidative damage markers such as MDA and 8‐OHdG. These observations align with our findings and support the protective effects of HES against VIN‐induced nephrotoxicity [30, 31].

Beyond oxidative stress, inflammatory and redox‐sensitive signaling pathways also contribute to VIN‐induced renal injury. In this context, our findings indicate that HES modulates these pathways, as evidenced by the upregulation of Nrf2 and HO‐1, alongside the downregulation of TNF‐α and IL‐1β. These findings are consistent with previous studies demonstrating that HES activates the Nrf2/HO‐1 axis while suppressing inflammatory mediators in experimental models of renal injury [32, 33].

In addition to oxidative and inflammatory alterations, the present findings indicate that VIN induces apoptosis through coordinated activation of mitochondrial and endoplasmic reticulum (ER) stress pathways. This is supported by the significant upregulation of p53, Bax, Caspase‐3, and CHOP expression, along with a concomitant decrease in Bcl‐2 levels, collectively indicating activation of both mitochondrial and ER stress‐mediated apoptotic pathways. The increase in Bax and reduction in Bcl‐2 suggest activation of the mitochondrial apoptotic pathway [34], while elevated Caspase‐3 confirms execution‐phase apoptosis [35]. In parallel, the upregulation of CHOP indicates the involvement of ER stress‐mediated apoptosis [36]. Importantly, HES supplementation markedly attenuated these alterations, as evidenced by the restoration of Bax/Bcl‐2 balance and the reduction of Caspase‐3 and CHOP expression levels, consistent with previous studies demonstrating that HES suppresses apoptosis by modulating Bax/Bcl‐2 signaling and inhibiting Caspase‐3 activation in experimental models of organ injury [37, 38, 39].

Consistent with the observed apoptotic alterations, VIN treatment significantly suppressed PI3K, Akt, and mTOR levels, indicating inhibition of a key pro‐survival signaling pathway [40, 41]. Given that the PI3K/Akt/mTOR axis plays a central role in regulating cell survival and negatively controlling autophagy, its downregulation likely contributed to the activation of stress‐induced autophagy in the present study [42, 43]. This is supported by the marked upregulation of autophagy‐related markers, including LC3B, ATG5, Beclin‐1, and ATG7, in the VIN‐treated group, which are widely recognized as key regulators of autophagosome formation and autophagic flux [44]. The inhibition of mTOR, a well‐established negative regulator of autophagy, is known to trigger autophagosome formation through activation of the ULK1–Beclin‐1 complex and downstream ATG proteins, thereby promoting autophagic activity [45, 46]. VIN has been reported to suppress the PI3K/Akt/mTOR signaling pathway, as evidenced by reduced mTOR activation, thereby contributing to decreased cell survival signaling and enhanced apoptotic responses [47]. Importantly, HES supplementation partially restored PI3K, Akt, and mTOR levels while attenuating the expression of autophagy‐related genes, suggesting that its protective effects may involve modulation of the PI3K/Akt/mTOR autophagy axis. This is consistent with previous studies demonstrating that HES activates PI3K/Akt/mTOR signaling, thereby suppressing excessive autophagy and preserving cellular integrity [48, 49]. To further substantiate the biochemical and molecular findings, histopathological evaluation was performed to assess the extent of structural renal damage. The histopathological findings obtained in the present study demonstrate that VIN administration induces severe renal damage characterized by marked tubular degeneration, epithelial cell loss, hyaline cast formation, and interstitial congestion. These findings are consistent with previous studies reporting that chemotherapeutic agents can induce nephrotoxic alterations in renal tissue [50, 51], and are further supported by the high damage scores observed in the semiquantitative evaluation employed in this study [24].

In contrast, the HES‐treated group exhibited a significant reduction in histopathological alterations, with substantial preservation of tubular architecture, regression of degenerative changes, and attenuation of interstitial congestion. Notably, the presence of only a limited number of atrophic tubules in the VIN + HES group indicates a marked histological improvement compared to the VIN group. These results are consistent with earlier research showing HES's ability to prevent chemotherapy‐induced nephrotoxicity [50, 52] and further support the histopathological recovery observed in the present study. A major strength of this study is the comprehensive evaluation of vincristine‐induced renal injury and the protective effects of hesperidin using complementary biochemical, molecular, and histopathological approaches. In particular, the simultaneous assessment of oxidative stress, inflammation, apoptosis, autophagy, and the PI3K/Akt/mTOR signaling pathway provides a broad mechanistic perspective on the renoprotective effects of hesperidin. Nevertheless, several limitations should be acknowledged. The study evaluated a single dose of hesperidin and included only female rats; therefore, potential sex‐dependent differences and dose‐response relationships could not be determined. In addition, although multiple signaling pathways were investigated, some molecular outcomes were primarily assessed at the expression level. Further functional and pathway‐specific studies may therefore be useful to validate the proposed mechanisms. These limitations should be considered when interpreting the broader and translational relevance of the findings.

## 5 Conclusions

Overall, this study shows that VIN damages the kidneys through a tangled web of interconnected problems: inflammation, oxidative stress, apoptosis, and messed‐up autophagy, along with disruption of the PI3K/Akt/mTOR pathways that normally help cells survive. Too much oxidative stress, specifically, an overload of ROS, seems to be the main trigger. It sets off a chain reaction that harms mitochondria, triggers stress in the endoplasmic reticulum, and shuts down pro‐survival signals while also cranking up stress‐induced autophagy. These molecular issues were backed up by clear changes in kidney function markers and visible tissue damage under the microscope. Importantly, giving HES helped reverse most of these problems. It restored the kidney's redox balance, reduced inflammation and cell death, normalized the PI3K/Akt/mTOR pathway, and brought excessive autophagy back under control, helping protect the integrity of kidney cells. The fact that all the biochemical, molecular, and tissue‐level findings line up so well really strengthens the case that HES works through multiple targets at once. Taken together, these results suggest that HES protects the kidneys by fine‐tuning a complex network involving redox balance, inflammation, apoptosis, and autophagy. It could be a promising strategy to reduce chemotherapy‐related kidney damage in patients.

## Author Contributions

Aydin Genc was responsible for the conceptualization and design of the study, drafted the initial manuscript, carried out critical revisions, and verified the data. Bayram Akoglan oversaw the research process, contributed software resources, managed project coordination, developed the methodology, conducted experiments, performed data analysis, and handled data management. Emre Sahin assisted in drafting and revising the manuscript, supported software and methodological aspects, participated in experimental work, contributed to supervision, and organized the dataset. Eren Cankaya conducted and evaluated the histopathological analyses. All authors reviewed and approved the final version of the manuscript for submission.

## Funding

The authors have nothing to report.

## Ethics Statement

The Animal Ethics Committee of Bingol University gave its approval to the animal protocol (Protocol No: E‐85680299‐020‐2600023988). Every animal experiment was conducted in compliance with the EU Directive 2010for animal research, the ARRIVE guidelines, and the U.K. Animals (Scientific Procedures) Act, 1986.

## Conflicts of Interest

The authors declare no conflicts of interest.

## Supporting information


Supporting File

## Data Availability

All data contributing to the findings of the present study are accessible via the corresponding author upon request.
