# Supplementary material for: Hesperidin Attenuates Vincristine‐Induced Nephrotoxicity via Nrf2/HO‐1 and PI3K/Akt/mTOR Signaling
Source: J Biochem Mol Toxicol. 2026 Oct 18;40(11):e71136. doi: 10.1002/jbt.71136 (PMC13676836; doi:10.1002/jbt.71136)
Supplement: Supplementary file 1 [file JBT-40-e71136-s001.pdf]

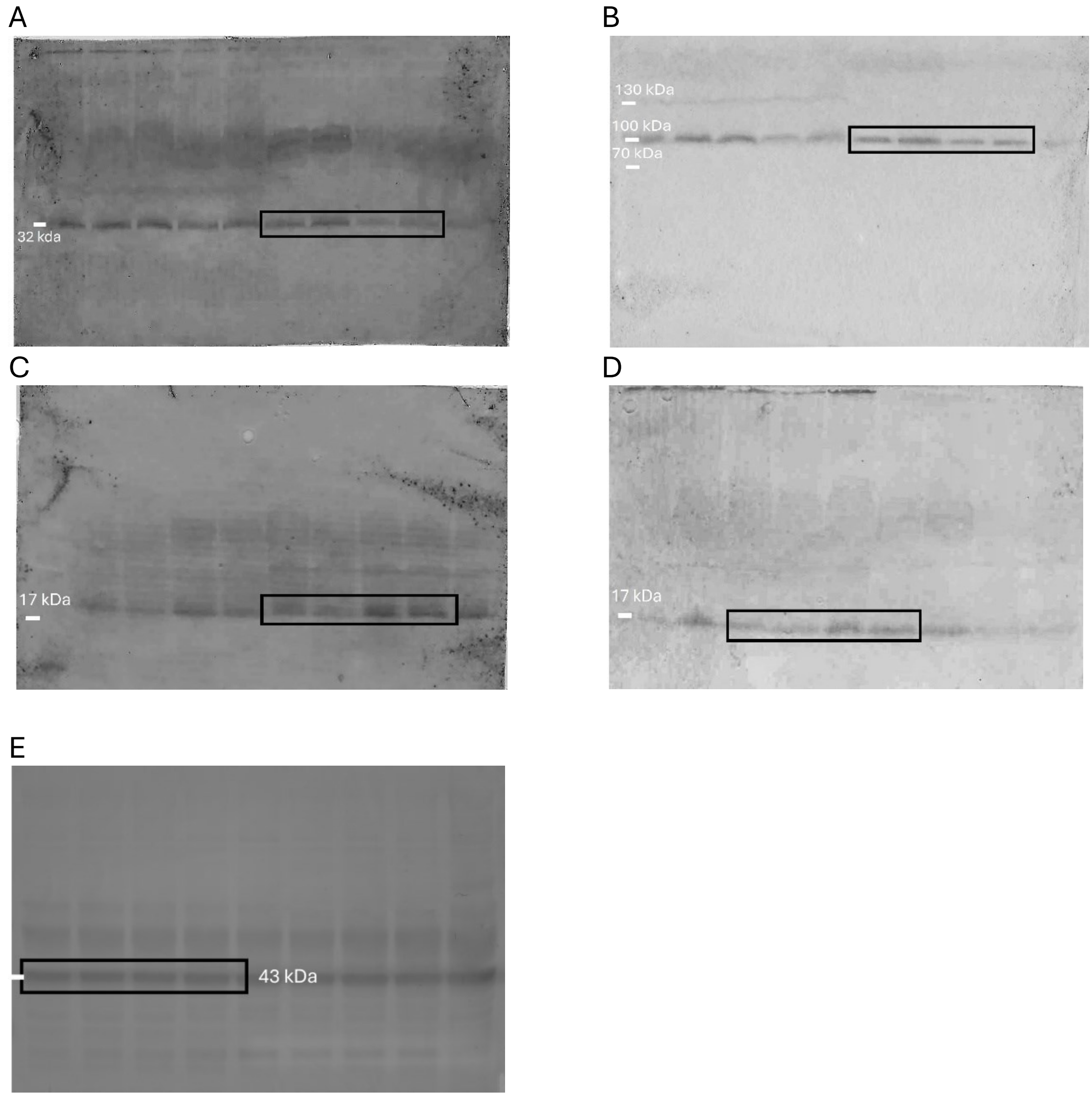

Figure S1. Full immunoblots corresponding to Figure 6 in the main text show representative Western blot images of the effects of silymarin supplementation on kidney protein levels of (A) HO-1, (B) Nrf2, (C) TNF-α, and (D) IL-1β in rats exposed to vincristine. Densitometric analysis of the relative band intensities was performed after normalization to (E) β-actin. Molecular weight (M.W.) markers are indicated in kilodaltons (kDa).
